# Lower limb muscle co-contraction and joint loading of flip-flops walking in male wearers

**DOI:** 10.1371/journal.pone.0193653

**Published:** 2018-03-21

**Authors:** Tony Lin-Wei Chen, Duo Wai-Chi Wong, Zhi Xu, Qitao Tan, Yan Wang, Ameersing Luximon, Ming Zhang

**Affiliations:** 1 Department of Biomedical Engineering, Faculty of Engineering, The Hong Kong Polytechnic University, Hong Kong SAR, China; 2 The Hong Kong Polytechnic University Shenzhen Research Institute, Shenzhen, Guangdong, China; 3 Laboratory of Biomechanical Engineering, Department of Applied Mechanics, Sichuan University, Chengdu, Sichuan, China; 4 Institute of Textiles and Clothing, The Hong Kong Polytechnic University, Hong Kong SAR, China; University of Illinois at Urbana-Champaign, UNITED STATES

## Abstract

Flip-flops may change walking gait pattern, increase muscle activity and joint loading, and predispose wearers to foot problems, despite that quantitative evidence is scarce. The purpose of this study was to examine the lower limb muscle co-contraction and joint contact force in flip-flops gait, and compare with those of barefoot and sports shoes walking. Ten healthy males were instructed to perform over-ground walking at self-selected speed under three footwear conditions: 1) barefoot, 2) sports shoes, and 3) thong-type flip-flops. Kinematic, kinetic and EMG data were collected and input to a musculoskeletal model to estimate muscle force and joint force. One-way repeated measures ANOVA was conducted to compare footwear conditions. It was hypothesized that flip-flops would induce muscle co-contraction and produce different gait kinematics and kinetics. Our results demonstrated that the musculoskeletal model estimation had a good temporal consistency with the measured EMG. Flip-flops produced significantly lower walking speed, higher ankle and subtalar joint range of motion, and higher shear ankle joint contact force than sports shoes (*p* < 0.05). There were no significant differences between flip-flops and barefoot conditions in terms of muscle co-contraction index, joint kinematics, and joint loading of the knee and ankle complex (*p* > 0.05). The variance in walking speed and footwear design may be the two major factors that resulted in the comparable joint biomechanics in flip-flops and barefoot walking. From this point of view, whether flip-flops gait is potentially harmful to foot health remains unclear. Given that shod walking is more common than barefoot walking on a daily basis, sports shoes with close-toe design may be a better footwear option than flip-flops for injury prevention due to its constraint on joint motion and loading.

## Introduction

Flip-flops are gaining popularity among people on a variety of occasions for their casual and comfortable style. A survey on 1000 females showed that approximately 43% of them preferred wearing flip-flops over sports shoes during shopping [1], while a fourfold increase in the sale of flip-flops to male customers was recorded by a market research firm from 2002 to 2006 [2]. Flip-flops typically feature a thin, flat, and flexible sole attached with few straps in the forefoot region [3]. Without the protection of heel counter that is commonly seen in the closed-toe shoes, flip-flops wearers largely expose their hindfoot and receive no supports to the heel and ankle due to the limited foot/shoes contacts [1,4]. This minimalist structure was speculated to cause many foot problems associated with mechanical instability [5]. A recent report on teenage population has attributed 37% of foot complaints and pain to the prolonged usage of flip-flops [6].

Previous walking trials showed that flip-flops reduced ankle plantarflexion at heel strike [7,8] and ankle dorsiflexion at toe-off [8] in comparison to athletic shoes. The reduced ankle range of motion (ROM) in flip-flops walking was thought to be a postural adjustment commonly adopted by the wearer to enhance foot/sole stability [1]. Besides, the ankle dorsiflexors and plantarflexors would co-contract to facilitate better control of joint motion [8]. Given that muscle force is the main contributor to joint loading [9,10], the increased muscular output in flip-flops walking may have adverse effects on foot health [11]. Excessive force across the joint is associated with the development of foot symptoms and disorders [12].

Some studies argued that the minimalism of flip-flops helped to simulate barefoot walking and might be safe to foot healthy on this basis [5]. Nevertheless, flip-flops were consistently reported to increase ankle dorsiflexion [4,6,8,13], knee flexion [4,6] and tibialis anterior activation [13,14] compared to barefoot walking. In combination, these changed gait patterns are likely to redistribute joint force and cause abnormal loading. Likewise, the altered loads on joint could lead to pain and a series of pathomechanical processes [15]. To this end, there is a sparsity of studies that comprehensively evaluates the lower limb muscle force and joint loading for flip-flops walking.

The recent development of computational simulation techniques provides a robust method to estimate lower extremity muscle and joint forces for various movement conditions [16,17]. These estimations permit a better understanding of the musculoskeletal demands for footwear-related gait alteration and the potential risks of foot injuries. The study sought to use musculoskeletal modeling to investigate the lower limb muscle activity and joint loading in flip-flop gait, and compare with those of sports shoes and barefoot walking. It was hypothesized that flip-flops would increase foot muscle co-contraction and peak knee/ankle joint contact force (JCF) during gait compared to other unshod/shod conditions.

## Methods

### Participants

Ten healthy males (age: 25.58 ± 3.64 years, height: 173.67 ± 1.52 cm, mass: 59.86 ± 3.80 kg) were recruited in this study. They were recruited from the university community and free of any lower limb musculoskeletal injuries. To facilitate footwear fitting and standardize inter-condition comparison, the foot size of each participant was measured using the Brannock device before the experiments. All participants reported that they were not regular flip-flops wearers and had never tried the experimental shoes before. They were fully informed of the research procedures and signed the written consent prior to the experiment. The study was approved by the Human Subjects Ethics Sub-Committee of the Hong Kong Polytechnic University (reference number: HSEARS20091216002).

### Equipment and procedure

Motion capture system with eight cameras (Vicon, Oxford Metrics Ltd., Oxford, United Kingdom) and four force platforms (OR6, AMTI, Watertown, United States) were used to measure marker trajectories and ground reaction forces. Pre-test camera calibration on the motion analysis system was completed with a residual error less than 0.3 mm in all sessions. A set of retroreflective markers was affixed to the participants. The maker set was configured based on the OpenSim full body model [18]. Briefly, the markers (diameter of 15 mm) were attached to the acromioclavicular joint, lateral/medial humeral epicondyles, radius/ulna styloid processes, posterior/anterior iliac spines, greater trochanters, lateral/medial femoral epicondyles, lateral/medial malleoli, calcaneal tuberosity and the base/head of the 1st and 5th metatarsals. To better track the lower limb segmental motion, three additional markers were attached to the lateral-anterior aspect of thigh and shank. Marker placement was conducted by the same investigator (TLC) throughout the experiments and firmly fixed to the body using medical glue. For the sports shoes condition, makers were placed on the skin through several cut-outs (with a diameter of around 36mm) on the corresponding vamp regions. Muscle excitation was measured by surface electromyography (EMG, BTS Engineering, Bolgona, Italy). The 8-mm Ag/AgCI electrodes were attached (with an inter-electrode spacing of 22 mm) onto the clean and shaven skin overlaying the rectus femoris, vastus lateralis, vastus medialis, medial hamstrings, lateral hamstrings, gastrocnemius lateralis, gastrocnemius medialis, tibialis anterior, and peroneus longus. The long head and short head biceps femoris were assumed to have the identical EMG pattern. Similar assumption was also applied to semimembranosus and semitendinosus. The EMG profile of vastus intermedius was defined as the mean between that of the vastus lateralis and vastus medialis. Electrode positioning was parallel to the muscle fiber direction over the mid-muscle belly as guided by the SENIAM [19]. The electromyography of the maximum voluntary contraction (MVC) of the foot muscles was measured using the method described by Soma and colleagues [20]. Participants were instructed to generate maximal force in corresponding function direction of the knee, ankle, and subtalar joints. One examiner applied resistant force to ensure the MVC value was obtained in the state of muscular isometric contraction. For those foot muscles from which surface EMG signals were infeasible to measure, the activation was estimated by OpenSim. No actuator constraints were applied to these muscles in the computed muscle control setting of OpenSim [21].

**Fig 1:**
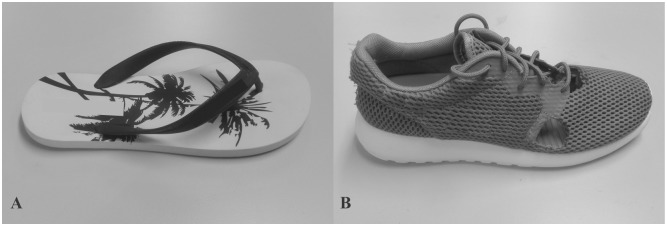
The footwear used in the experiment. (A) Thong-style flip-flops. (B) Sports shoes. Some regions of the vamp were drilled into openings (with a diameter of 3–3.5cm) for marker installment.

The participants were asked to walk over-ground at their self-selected speed in three different footwear conditions: barefoot, sports shoes (Roshe Run, Nike Inc., Oregon, United States), and flip-flops (Flipper, Adidas, Herzogenaurach, Germany). The flip-flops used in this study were a thong style slipper with a Y-shaped strap loosely held on the footbed. The sole was made of rubber and flat-shaped (Fig 1). The sports shoes had simplified-designed vamp and outsole structures that provided limited supports and cushioning for the foot. The shoe models were selected because they are common in daily life and present the major features of the respective categories. The footwear conditions were randomized and the participants had about five minutes to familiarize themselves with the shoes. Prior to dynamic trials, the participants were asked to stand still within the capture volume and a static trial was performed for each footwear condition. The participants then conducted six walking trials on a 10m pathway. They were instructed to trace a straight line and avoid targeting the force platform when walking over it. A trial was considered valid when (1) the participant’s footstep entirely fell within the force platform and (2) there was no attempted alteration in the walking style as judged subjectively by the investigator.

### Data collection and processing

The sampling frequencies of the motion capture system, force platform, and electromyography were 100 Hz, 1000 Hz, and 1000 Hz respectively. All data were collected synchronously through the Vicon system, Kinematics and kinetics data were filtered using a fourth-order, phase-corrected, Butterworth, low-pass filter at 8 Hz and 50 Hz respectively. The raw EMG data were band-pass filtered (10–450 Hz), full-wave rectified and then low-pass filtered at the cutoff frequency of 6 Hz. The resulting linear envelopes were amplitude-normalized to the maximal EMG value of each muscle obtained from the MVC tests and then time-normalized to 100% gait cycle [22]. The level of muscle co-contraction was determined by the co-contraction index (CCI) for each data point of the normalized linear envelopes. The equation for calculating the CCI is displayed as following [23,24]:

$${CCI}_{i=1}^{n}=\sum_{i=1}^{n}\frac{{lowEMG}_{i}}{{highEMG}_{i}}\times ({highEMG}_{i}+{lowEMG}_{i})$$ (1)

Where the *lowEMG*_*i*_ and *highEMG*_*i*_ are the normalized EMG value of the co-contracted muscles in the pair of co-contraction (*low* represents the less active muscle and *high* is the higher active one), with *i* denoting one data point in the EMG envelope. *n* represents the number of data points in the stance phase. For the sake of standardization, *n* was set to 63 for all three experimental conditions (the averaged stance phase across the conditions was 63.52% and the gait cycle for all subjects were scaled to contain 100 data points). The method gave an estimate of the magnitude of co-contraction based on the relative activation of the muscle pair (S1 File) [23]. In the study, the following pairs of muscles were investigated [25]: vastus lateralis and gastrocnemius medialis (VL/GM), vastus lateralis and biceps femoris (VL/BF), vastus medialis and gastrocnemius medialis (VM/GM), vastus medialis and biceps femoris (VM/BF), gastrocnemius medialis and tibialis anterior (GM/TA), and peroneus longus and tibialis anterior (PL/TA). These pairs of muscles were selected because of their functions in stabilizing knee, ankle, and subtalar joints. The resulting CCI time-series for the stance phase (identified by vertical ground reaction force above 10 N) was calculated and averaged across the three walking trials for each participant. Data process was conducted using customized MATLAB scripts (The MathWorks Inc., Ismaning, GER).

### Musculoskeletal modeling

Data of maker trajectory and ground reaction force was converted and input to drive a musculoskeletal model using the OpenSim platform (National Center for Simulation in Rehabilitation Research, Stanford, United States). The generic model [18] featured 22 rigid-body segments, 37 degrees of freedom and 80 musculotendonous units. The hips were modeled as ball-and-socket joints, and the knees was modeled as hinge joints. The ankle, subtalar and metatarsophalangeal joints were modeled as revolute joints. The model was firstly scaled using the data of the static trial to accommodate the participant’s anthropometry. The inverse kinematics was solved for joint kinematics that minimized the trajectory differences between experimental markers and virtual markers. Dynamic inconsistency was reduced by small adjustments to model mass properties and kinematics [17]. Muscle force was estimated using computed muscle control [26]. The processed EMG envelopes were input as the constraint for muscle activation. The range of the activation was initially outlined as ±5% of the normalized EMG value. The constraint would be softened at the increasing interval of 5% until simulation convergence obtained [27]. Knee and ankle JCF was calculated as the sum of joint reaction force and muscle force that spanning the joint [28]. The direction of muscle force corresponding to the segmental frame was obtained using an OpenSim plugin [29]. Peak JCF during the stance phase was determined and averaged across three trials for each participant.

### Statistical analyses

Statistics analysis was performed in SPSS (SPSS, Inc., Chicago, IL, USA). The data were reported in mean and standard deviation (SD, the underlying data set is in S2 File). A one-way repeated measures ANOVA was conducted to examine the influence of footwear conditions on general kinematic parameters, CCI and peak JCF, followed by post-hoc pairwise comparison using LSD correction if significance exists. The significant level was set at 0.05. A pre-hoc test demonstrated that all data were normally distributed, as assessed by Shapiro-Wilk’s test (*p* > 0.05). The temporal similarity between the normalized EMG envelope and corresponding simulated muscle activation was assessed by cross-correlation analysis [30] and linear regression analysis [31]. Since the two waveforms were all scaled to percentile gait cycle (with 100 data points each). The cross-correlation sequence *R* for two curves with zero-time lag was calculated as:

$$R=\frac{\sum x_{i}y_{i}}{{\left(\sum x_{i}^{2}\right)}^{1/2}{\left(\sum y_{i}^{2}\right)}^{1/2}}$$ (2)

Where the *x*_*i*_ and *y*_*i*_ were the values of the two curves at data point *i*. *R*-value ranges from 0 to 1 (with 1 indicating that the two curves have the exactly same shape) and is sensitive to the similarity in timing [22].

## Results

### General gait characteristics

As shown in Table 1, the walking velocity was significantly different among the experimental conditions [*F* (2, 18) = 10.02, *p* < 0.05]. Pairwise comparison revealed that flip-flops had a lower walking velocity than that of the sports shoes (*p* < 0.01). Walking velocity of flip-flops and barefoot conditions was comparable (*p* > 0.05). Besides, flip-flops conditions presented a slower cadence and shortened stance phase, despite significance could not be achieved (*p* > 0.05). In terms of joint kinematics, significant differences were reported in ankle [*F* (2, 18) = 6.73, *p* < 0.05] and subtalar joints [*F* (2, 18) = 4.45, *p* < 0.05]. Flip-flops produced higher ankle and subtalar ROM than sports shoes (*p* = 0.019–0.041). Other than that, all variables of joint motion were similar between flip-flops and barefoot conditions.

**Table 1:** Kinematic parameters and joint contact force.

Variables		Barefoot	Sports shoes	Flip-flops	*p*-value
Velocity (m/s)		1.25 (0.08)	1.31 (0.13)^1^^,^^3^	1.21 (0.10)	**0.001**
Cadence (steps/min)		59.76 (4.63)	58.15 (3.90)	56.50 (3.57)	0.201
Stance phase (%)		65.05 (6.32)	63.51 (1.68)	62.01 (2.16)	0.338
Joint range of motion (degrees)	Knee	50.33 (4.57)	50.76 (5.34)	51.17 (5.87)	0.808
	Ankle	21.82 (3.94)^2^	19.67 (3.92)^1^^,^^3^	21.78 (3.32)^2^	**0.007**
	Subtalar	11.73 (1.22)	10.86 (2.63)^3^	12.62 (2.05)^2^	**0.027**
Knee JCF (BW)	Compression	5.79 (1.72)	5.36 (1.37)	5.49 (2.11)	0.608
	Shear	0.89 (0.29)	0.99 (0.42)	0.95 (0.35)	0.586
Ankle JCF (BW)	Compression	6.07 (1.15)	5.45 (0.99)	5.64 (0.85)	0.063
	Shear	1.33 (0.61)^2^	0.89 (0.46)^1^^,^^3^	1.17 (0.46)^2^	**0.011**

BW, body weight; *p*-value less than or equal to 0.05 is bold; The numeric superscript indicates the groups between which there were statistically significant differences in the pairwise comparison.

^1^Compared to barefoot condition.

^2^Compared to sports shoes condition.

^3^Compared to flip-flops condition.

### Muscle co-contraction

**Fig 2:**
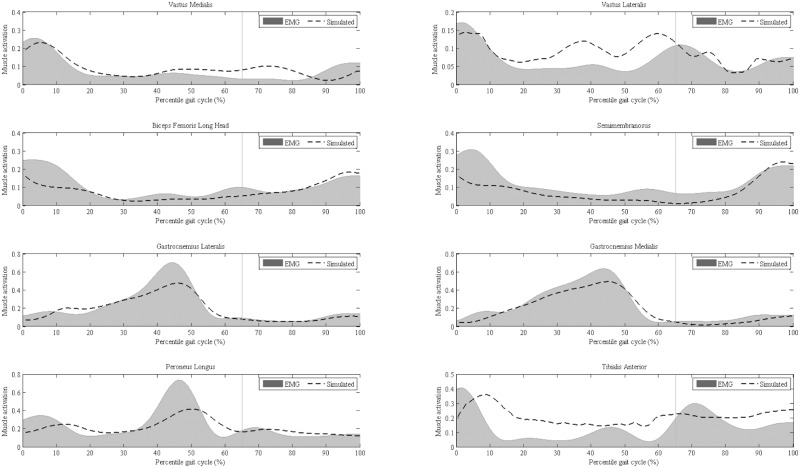
Simulated muscle activations compared to measured EMG for the lower limb muscles (starting from heel strike to the end of swing phase). The vertical solid line denotes the timing of toe-off.

Fig 2 plots the normalized EMG envelope and simulated muscle activation for selected muscles. The cross-correlation sequences show a good timing of the simulation in tracking the experimental muscular activity during normal walking (*R* = 0.865–0.988 for all pairs of comparison) [25]. Linear regression analysis reported the Pearson correlation coefficient of 0.85–0.99 (slope: 0.98–1.12, intercept: 0.0005–0.0080), indicating a good agreement between the EMG measurements and model estimation. Some notable differences in the magnitude of activation were presented in vastus lateralis and tibialis anterior from the mid to late stance phase. As pinpointed by the OpenSim developer [18], muscle output in the simulation could be increased to counteract the residual force generated by passive stretching of the knee flexors and ankle plantarflexors. The results of CCI for the selected muscle groups are displayed in Table 2. No significant differences were reported by the statistics in any pair of the comparisons. Overall, the magnitude of CCI calculated in the study was lower than that of previous research [25]. Horsak et al. used the peak muscle activity from walking tria0ls to normalize the EMG envelope, while we measured EMG signals during MVC tests for the targeted muscle groups, which is likely to induce higher muscle activity and result in lower normalized EMG values.

**Table 2:** CCI for the select muscle groups.

	Barefoot	Sports shoes	Flip-flops	*p*-value
VL/GM	5.23 (2.60)	4.96 (3.09)	5.21 (3.40)	0.895
VL/BF	5.21 (2.89)	6.08 (3.62)	5.41 (2.85)	0.281
VM/GM	4.63 (3.77)	4.01 (2.80)	5.02 (4.72)	0.250
VM/BF	4.72 (4.72)	4.91 (3.95)	5.84 (5.60)	0.173
GM/TA	6.19 (2.53)	5.96 (2.05)	6.54 (2.85)	0.323
PL/TA	9.27 (4.57)	8.92 (2.87)	9.19 (4.30)	0.891

### Joint contact force

There were no significant differences in knee JCF among the footwear conditions, as shown in Table 1. In terms of the ankle joint, significance was reached in the comparison of shear ankle JCF [F (2, 18) = 5.93, *p* < 0.01]. The post-hoc test showed that both barefoot and flip-flops had higher shear ankle JCF than the sports shoes (*p* = 0.027–0.048). Likewise, the shear ankle JCF was similar between the flip-flops and barefoot conditions (*p* > 0.05).

## Discussion

The aim of the study was to examine the lower limb muscle activity and joint loading during gait in flip-flops, and compare with barefoot and sports shoes walking. The results suggested that participants in flip-flops walked relatively slower and exhibited increased ankle/subtalar ROM and shear ankle JCF in comparison to sports shoes. However, flip-flops did not enhance foot muscle co-contraction for the wearers. In addition, the majority of the outcomes were similar between the flip-flops and barefoot conditions, including CCI and JCF.

It was hypothesized that flip-flops would induce higher co-contraction of the muscles that span the knee and ankle joints. Muscle co-contraction was thought to restrict excessive foot movements and regulate the lower limb joints as compensation for the unstable foot/sole interface of flip-flops. Nevertheless, previous research also reported conflicting results regarding the effects of unstable footwear on muscle activity. Some studies showed that wearing flip-flops [32] and shoes with unstable sole [33,34] had no effects on foot muscle EMG output, while others presented opposite findings [35]. Except for the differences in methodology for analysis and lab setting, one major factor that can influence muscle activity is the walking speed [36,37]. It was reported that young participants were sensitive to gait velocity and exerted increased antagonist activation in accelerating walking [38]. The present study did not standardize walking speed for the participants. Given that walking speed was significantly lower in the flip-flops condition, this might even out the possible increases in foot muscle co-contraction for flip-flops gait. In fact, reduced walking speed was consistently observed in flip-flops wearers [4,7,8,39]. It could be argued that if slow walking is the inherent nature of wearing flip-flops, then perhaps controlling it in the experiment limits the external validity of the results. On a daily basis, it is not necessary for the wearer to maintain the same speed level when walking in different footwear. Instead of invoking muscle co-contraction, reducing speed is seemly a more natural approach for the wearers to avoid injuries associated with footwear instability. Another possible explanation for insignificant differences in CCI might be that, the instability introduced by flip-flops was relatively small to trigger the neuromuscular and co-contraction response. In fact, flip-flops demonstrated higher CCI in GM/TA and PL/TA antagonism than sports shoes, though significance was not reached. The influence of footwear design elements was not considered in this study and it could be the reason for the variations. Flip-flops with different stiffness and structures were reported to attenuate foot stability differently [7]. Besides, there was significantly higher ankle and subtalar ROM in flip-flops compared to that of sports shoes [40]. The results were in partial accordance with those of previous studies [4,6,13]. The lower ROM but similar muscular control for sports shoe could be attributed to its close-toe design and the structural stiffness of the footwear. Conversely, flip-flops demanded a higher range of ankle motion to maintain the adherence between the sole and the foot due to a lack of shoe straps [4]. It is unclear to what extent this increased joint ROM would affect foot health. However, previous studies have associated excessive foot joint motion with injuries, most commonly related to overloaded ligaments spanning the joint and tissue rupture [41,42]. Given the fact that walking environment could vary greatly in real life, the ankle/subtalar ROM in flip-flops gait is likely to increase when the wearers need to walk faster [43]. From this point of view, shoes that give a certain constraint on foot joint movement, e.g., sports shoes, may be the better footwear for injury prevention [43,44].

Joint force was frequently assessed for the lower limb joints in many studies [9,12,45]. The magnitude of knee and ankle JCF in our study was relatively higher than those reported in walking. The reason could be that EMG signal was supplemented to the modeling in our study to account for muscle physiology and co-contraction, while some other studies may underestimate the joint loading based on a purely mathematical solution [46]. Our results showed that JCF was similar across the three conditions, except that peak ankle JCF in the shear direction was higher in flip-flops than sports shoes. Since foot muscle co-contraction was barely influenced by the footwear conditions, we speculated that the force line of ankle joint loading was resolved more into the transverse plane (shear direction) due to the increased ankle and subtalar ROM in flip-flops gait. Higher shear JCF could be of concern for flip-flops walking, given that lateral forces are not well tolerated by cartilage and bones [47]. In contemporary society, barefoot walking is not a popular phenomenon since people need the protection of plantar foot from the walking surface. Shod walking is a necessity for both life and work. On this basis, shoes with close-toe design could be the healthier footwear for injury prevention because it produced lower joint loading than flip-flops. Some researchers argued that minimalist footwear increased the sensory input from the thin sole [48] and facilitated the neural reflexes to the changed walking surface [49]. Nevertheless, normal sports shoes were seldom reported to jeopardize motor control for healthy users. In this study, the smaller shear force on ankle joint in sports shoes condition may represent a protective lower limb alignment as a result of neuromuscular adjustments.

There were some limitations that should be acknowledged. Only male participants were included in this study, whereas female participants were believed to have different anthropometry and alignment features that affected their walking biomechanics [50]. Besides that, the participants were inexperienced flip-flops users at the onset of our study. Novice wearers of one footwear type could respond differently in walking kinematics pre- and post-training session. Foot joint motion was commonly reduced after an accommodation period [34,35]. This could be another factor that influenced our research outcomes. The results of the present study might only pertain to the immediate effects of wearing flip-flops for the wearers having little experiences with the shoes. Finally, though the generic model was scaled to participant’s anthropometry in the study, individualized muscle morphology and property was not taken into account in the simulation. Further study was suggested to develop models with adjustable muscle parameters that can account for the subject-specific physiology. Moreover, musculoskeletal model assumes that the influence of cartilage and encapsulated soft tissue is negligible. The stress and deformation of foot bones and soft tissue can be further investigated using finite element method [51,52].

## Conclusions

Despite that flip-flops produced significantly lower walking speed, higher ankle and subtalar joint range of motion and higher shear ankle joint contact force than sports shoes (*p* < 0.05), there was no significant difference in muscle co-contraction index, joint kinematics and joint loading of the knee and ankle complex compared to barefoot condition (*p* > 0.05). The comparable results of joint biomechanics in flip-flops and barefoot walking could be attributed to the variance in walking speed and footwear design across conditions. It is thus difficult to conclude the effects of flip-flops gait on foot health based on the research outcomes. In the view of better injury prevention during shod walking, sports shoes with close-toe design would be preferable to constrain joint motion and loading compared to flip-flops.

## Supporting information

S1 File Example of the calculation procedure of CCI for one representative subject. (DOCX)

S2 File The data set of kinematic parameters, joint motion, muscle co-contraction index, and lower limb joint contact force for the individual participant. (XLSX)
