# Supplementary material for: Lower limb muscle co-contraction and joint loading of flip-flops walking in male wearers
Source: PLoS One. 2018 Mar 21;13(3):e0193653. doi: 10.1371/journal.pone.0193653 (PMC5862437; doi:10.1371/journal.pone.0193653)
Supplement: S1 File — (DOCX) [file pone.0193653.s001.docx]

**Example of the calculation procedure of CCI for one representative subject**


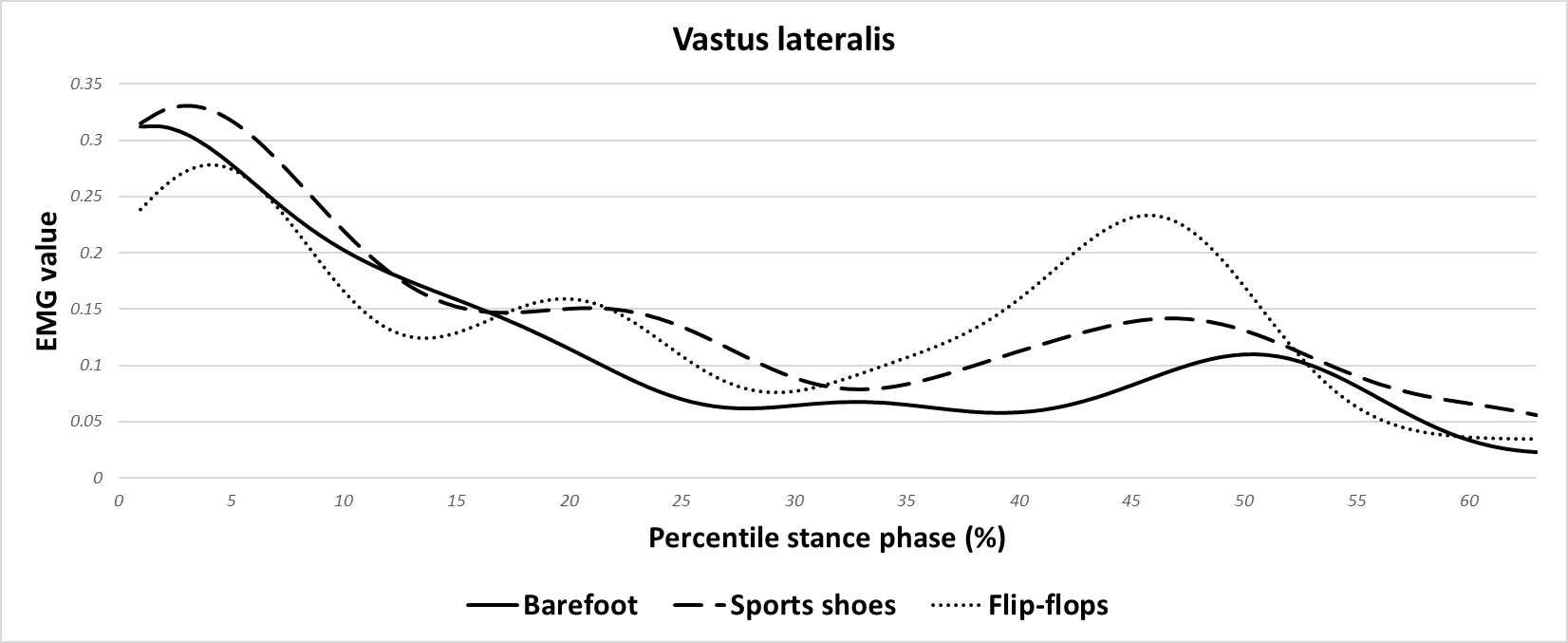

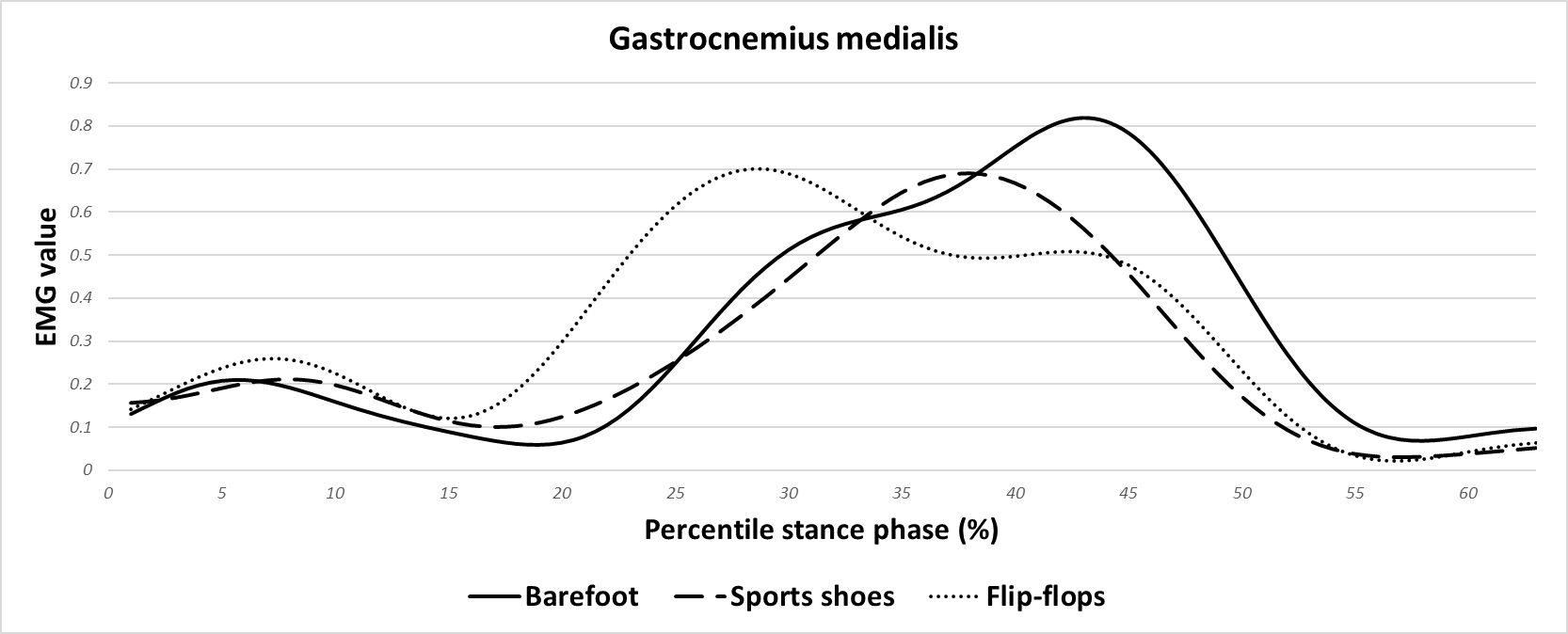
The following figures (one representative subject) show the normalized EMG value of VL and GM in percentile stance phase. Data size of one gait cycle was scaled to 100 and thus the stance phase contained 63 data points (stance phase averagely accounts for 63% of the gait cycle, as we elaborated in the manuscript).

To take the first data point of the barefoot condition as an example, EMG value of the VL (0.312) and GM (0.130) at this data point was put into the equation:

$${CCI}_{i=1}=\frac{{lowEMG}_{i}}{{highEMG}_{i}}\times({highEMG}_{i}+{lowEMG}_{i})$$

*i* was the data point (in this case *i* was the first data point). *lowEMG_i_* was the EMG value of GM (at first data point the EMG value of GM was lower than that of VL). *highEMG_i_* was the EMG value of VL. Therefore, *CCI_i=1_* equaled to 0.19.

By summing up the *CCI* value of all 63 data points in the stance phase, we obtained a resulting *CCI^n=63^* of 8.51 for the barefoot condition. Resulting *CCI^n=63^* for the other two conditions was calculated in the same manner (11.48 for sports shoes condition and 14.43 for flip-flops condition).
